# Case Report: C-Reactive Protein Apheresis in a Patient With COVID-19 and Fulminant CRP Increase

**DOI:** 10.3389/fimmu.2021.708101

**Published:** 2021-08-02

**Authors:** Jens Ringel, Anja Ramlow, Christopher Bock, Ahmed Sheriff

**Affiliations:** ^1^Nephrology, DIAMEDIKUM, Potsdam, Germany; ^2^Apheresis Unit, Pentracor GmbH, Hennigsdorf, Germany; ^3^Gastroenterology/Infectiology/Rheumatology, Charité University Medicine Berlin, Berlin, Germany

**Keywords:** COVID-19, C-reactive protein, SARS-CoV-2, apheresis - therapeutic, pulmonary fibrosis (MeSH)

## Abstract

**Background:**

Plasma levels of C-reactive protein (CRP), induced by Severe Acute Respiratory Syndrome Coronavirus-2 (SARS-CoV-2) triggering COVID-19, can rise surprisingly high. The increase of the CRP concentration as well as a certain threshold concentration of CRP are indicative of clinical deterioration to artificial ventilation. In COVID-19, virus-induced lung injury and the subsequent massive onset of inflammation often drives pulmonary fibrosis. Fibrosis of the lung usually proceeds as sequela to a severe course of COVID-19 and its consequences only show months later. CRP-mediated complement- and macrophage activation is suspected to be the main driver of pulmonary fibrosis and subsequent organ failure in COVID-19. Recently, CRP apheresis was introduced to selectively remove CRP from human blood plasma.

**Case Report:**

A 53-year-old, SARS-CoV-2 positive, male patient with the risk factor diabetes type 2 was referred with dyspnea, fever and fulminant increase of CRP. The patient’s lungs already showed a pattern enhancement as an early sign of incipient pneumonia. The oxygen saturation of the blood was ≤ 89%. CRP apheresis using the selective CRP adsorber (PentraSorb^®^ CRP) was started immediately. CRP apheresis was performed *via* peripheral venous access on 4 successive days. CRP concentrations before CRP apheresis ranged from 47 to 133 mg/l. The removal of CRP was very effective with up to 79% depletion within one apheresis session and 1.2 to 2.14 plasma volumes were processed in each session. No apheresis-associated side effects were observed. It was at no point necessary to transfer the patient to the Intensive Care Unit or to intubate him due to respiratory failure. 10 days after the first positive SARS-CoV-2 test, CRP levels stayed below 20 mg/l and the patient no longer exhibited fever. Fourteen days after the first positive SARS-CoV-2 test, the lungs showed no sign of pneumonia on X-ray.

**Conclusion:**

This is the first report on CRP apheresis in an early COVID-19 patient with fulminant CRP increase. Despite a poor prognosis due to his diabetes and biomarker profile, the patient was not ventilated, and the onset of pneumonia was reverted.

## Introduction

Coronavirus disease 2019 (COVID-19) is caused by severe acute respiratory syndrome coronavirus 2 (SARS-CoV-2) and has evolved into a pandemic causing a public health crisis (1, 2). It is diagnosed by detection of virus in respiratory secretions using PCR analysis. The course of disease is mild in the majority of patients with symptoms including sore throat, cough, breathlessness, fever, and fatigue among others. In some patients, however, the disease may progress to viral pneumonia and acute respiratory distress syndrome (ARDS), finally leading to multi-organ failure. In this small percentage, SARS-CoV-2 induces lung fibrosis and cardiac complications (3–5). So far, primary therapy is symptomatic and focuses on ARDS. Given it is a viral disease, plasma levels of C-reactive protein (CRP), a common bacterial infection marker, are surprisingly high. Further, CRP levels were consistently shown to correlate with the severity of the disease, the overall clinical situation and the risk for the patient to need mechanical ventilation (6–13).

CRP is an ancient antibody, being part of the innate immune response and increasing rapidly during the acute phase reaction. Increasing evidence suggests that CRP causally promotes tissue destruction during the inflammatory response under certain pathological circumstances (14). Intraalveolar edema and hemorrhage are a common observation in the lungs of COVID-19 patients, resulting in ischemic alveolar tissue. CRP itself is reported to trigger tissue damage by binding to these ischemic cells and thus is also causally involved in the enlargement of the destroyed tissue, contributing to irreversible tissue destruction (14). Two well-described immunological CRP functions are the activation of the classical complement pathway *via* C1q binding (15) and the binding to human immunoglobulin Fc*γ*-receptors (mainly Fc*γ*RIIa) after opsonization of biological particles for macrophages (16–18). The opsonization of affected endogenous cells significantly contributes to the enlargement of organ damage (19). In the case of progressive, severe COVID-19 with high CRP plasma concentrations, pulmonary tissue is irreversibly disposed by the action of CRP (20).

So far, there is no pharmaceutical option to reduce the extremely high synthesis/level of CRP during an acute phase response, but the rapid reduction of high CRP levels in medium and severe courses of COVID-19 was proposed early on during the pandemic (21–23) and also recently (14, 24).

Selective, extracorporeal CRP apheresis with a CRP adsorber (25) lowers the CRP concentration drastically within a few hours, and the repeatable treatment is safe and efficient (26, 27). Clear evidence has been shown in previous clinical studies, investigating CRP apheresis after myocardial infarction, that CRP depletion reduces systemic inflammation and cardiac tissue damage (27). We used this therapy in the early phase of incipient pulmonary fibrosis in a patient diagnosed with SARS-CoV-2 infection.

## Case Report

### Patient Information

A 53-year-old male Caucasian with diabetes type 2 was tested positive for SARS-CoV-2 with a rapid antigen test. Diagnosis was substantiated with PCR testing afterwards. Moderate symptoms started roughly 48 h after the first positive test, including increasing fever, shivering, listlessness, hyperhidrosis, lack of exercise tolerance, mild hypoxia, shallow respiration and severe coughing. The patient was first examined 5 days after the positive antigen test. He showed an overall poor physical condition. Oxygen saturation was measured continuously and fluctuated above 90%, however it momentarily reached 89%, which led to referral to a chest x-ray. As the oxygen saturation improved during the first day of treatment, possibly due to rehydration, no oxygen supplementation was performed. Symptoms and CRP plasma levels were monitored twice daily. 5 days after the initial positive test, CRP concentrations increased rapidly. Based on the rate of increase in CRP concentration, which is a prognostic marker for the potential onset of ventilation requirement (7), treatment with CRP apheresis was decided. First CRP apheresis was initiated 5 days after positive SARS-CoV-2 test (3 days after symptom onset) and four apheresis treatments were performed on consecutive days with the PentraSorb CRP adsorber (**Table 1**).

**Table 1:** Timeline of episode of care.

Day 0	SARS-CoV-2 positive after rapid antigen test Regular domestic quarantine and daily monitoring of CRP plasma concentration and symptoms Difficulty sleeping
Day 2	Coughing irritation when trying to take a deep breath, coughing hurts
Day 3	Coughing persists
Day 4	Coughing for several hours, difficulty breathing without coughing irritation 2 hours of chills including severe sweating -> dehydration Performance slump
Day 5 Start of treatment 1^st^ apheresis	The positive test result of the PCR from Day 0 is available Rapid CRP concentration increase on the two previous days Overall poor physical condition, fever Oxygen saturation of the blood was 89% Venous, typically dark, hypoxemic blood Dehydration with a picture of mild coagulopathy Fluid substitution with 250 ml NaCl before and during apheresis, nevertheless only low plasma flow (20 mL/min)
Day 6 2^nd^ apheresis	Thorax X-ray Fluid substitution with 100 ml CaCl_2_ and 500 ml NaCl before apheresis; 250 ml NaCl during apheresis Overall poor physical condition, fever Patient received additional heparin starting halfway through apheresis
Day 7 3^rd^ apheresis	300 ml NaCl during apheresis Patient is negative in Corona rapid test 6 mg dexamethasone as a pill for 7 days starting 2 h after apheresis
Day 8 4^th^ apheresis End of treatment	Patient is more agile and subjectively fitter than on the previous days 500 ml NaCl during apheresis Deep sleep at night for the first time again with preserved hyperhidrosis
Day 9	Breathing improves to about 2/3 of normal
Day 10	Patient feels fit and can breathe deeply again 8 kg weight loss = 11% of body weight
Day 14	Follow-up thorax X-ray
Day 35	Transthoracic Echocardiography Resting Echocardiography
Day 49	Cardiac magnetic resonance imaging

Of note, a bacterial infection was excluded by measuring Procalcitonin (0.22 µg/L 3 days after symptom onset).

### Treatment and Outcome

CRP concentration kinetics showed a rapid increase in plasma levels between 96 and 132 h after positive SARS-CoV-2 antigen test and 1^st^ apheresis was then initiated. Apheresis treatments were performed as described elsewhere in detail (27) and the blood was anticoagulated with ACD-A (1^st^ apheresis) or ACD-A and additional heparin (2^nd^ - 4^th^ apheresis). Treated plasma volume was dependent on patient condition and CRP concentration. Apheresis parameters are listed in **Table 2**.

**Table 2:** Apheresis specifications.

	1^st^ apheresis	2^nd^ apheresis	3^rd^ apheresis	4^th^ apheresis
Time between positive test and start of apheresis [h]	125	148	170	192
Duration of treatment [h]	3.8	4.9	7	4.2
Processed Plasma [ml]	5500	3520	7000	4000
Plasma volume	1.84	1.29	2.14	1.22
CRP level at start of apheresis [mg/l]	47	106	133	61
CRP level at end of apheresis [mg/l]	33	50	35	13
CRP depletion [%]	30	53	75	79

**Figure 1:**
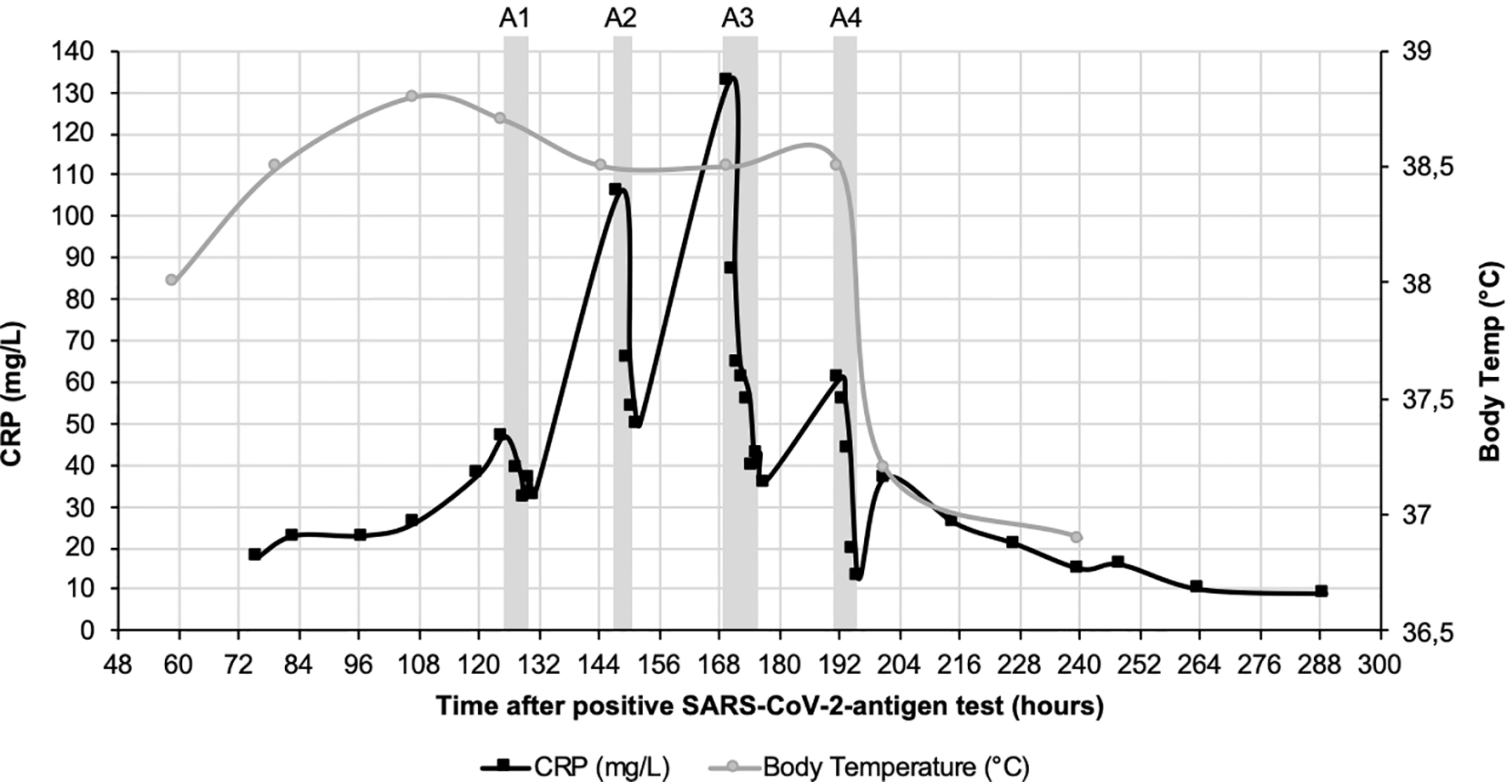
CRP course (black) and course of body temperature (grey) over 12 days after positive SARS-CoV-2-antigen test. CRP concentration was measured in plasma. Fever decreased 8 days after positive SARS-CoV-2 test during the last CRP apheresis. Grey boxes indicate the four apheresis treatments (A1-4).

CRP plasma concentrations declined rapidly during apheresis with a maximal depletion of 79% during the last apheresis session (**Figure 1**). Maximum CRP concentration of 133 mg/l was reached 7 days after positive antigen test and before 3^rd^ apheresis. Massive CRP synthesis was observed during the first two CRP aphereses. CRP levels stayed below 50 mg/l after the fourth apheresis treatment and normalized (< 10 mg/l) 12 days after the first positive antigen test.

Body temperature was measured daily and stayed consistently above 38.0°C until 8 days after positive antigen testing, when it decreased to a physiological 37.2°C during the last CRP apheresis, simultaneously reflecting the better condition of the patient (**Figure 1**).

During apheresis treatments, general cardiovascular parameters were measured at least hourly and did not show severe abnormalities. Blood pressure and pulse remained stable albeit the pulse was elevated over all four treatments. Sodium, Potassium, Calcium, Lactate and Glucose measurements showed normal concentrations without large changes during apheresis sessions. Venous O_2_ saturation improved during all four treatments. The patient’s hematocrit was influenced by fluid substitution during aphereses and decreased slightly during the treatments.

**Figure 2:**
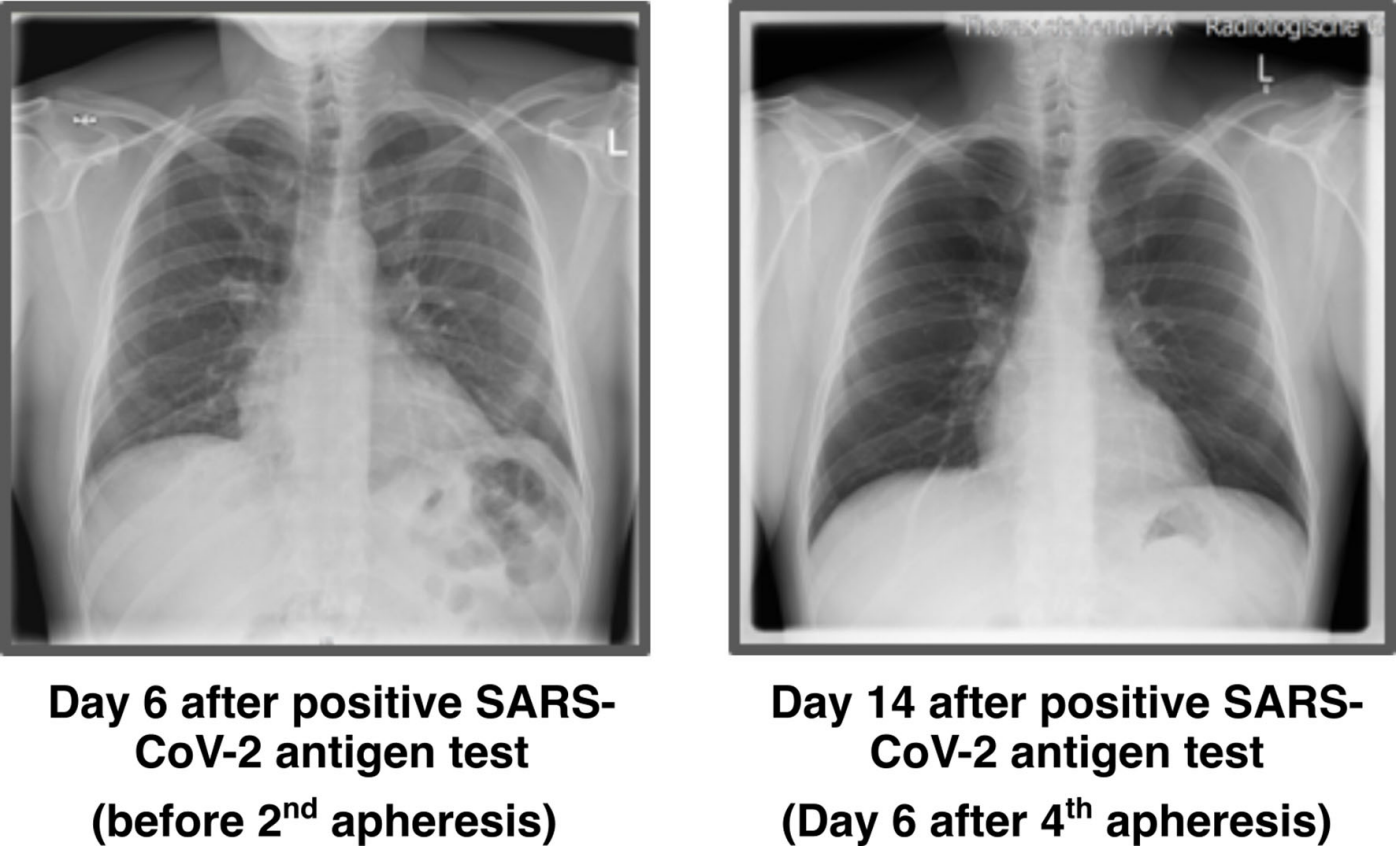
Chest X-ray images. X-ray thorax in 2 planes left adjacent. 6 days: Streaky compressions of the lung in the lower field/retrocardial left side. A low-grade concomitant effusion on the left. Heart normal size, no congestion. Upper mediastinum slender. No pneumothorax. Bony thorax intact. 14 days: No compressions and effusions.

Chest X-ray was performed before 1^st^ apheresis (day 5 after positive antigen test) and showed streaky compressions of the lung in the lower field/retrocardial left side. The abnormalities were consistent with the expected pattern of atypical pneumonia. The 2^nd^ X-ray was performed 6 days after the last apheresis session and showed no more pathological findings (**Figure 2**).

Transthoracic Echocardiography was performed 27 days after last apheresis session with sinus rhythm. It exhibited no pathological findings. Left and right ventricle displayed normal size and healthy systolic and diastolic function. No hypertrophy or pericardial effusion was detected. Both atriums displayed normal size and cardiac valves exhibited regular function. Resting echocardiography showed no pathological findings as well.

Cardiac MRI was performed 41 days after last apheresis session and showed normal, healthy cardiac function. Specifically, there was no indication for exercise-induced myocardial ischemia, cardiomyopathy, acute/chronic peri/myocarditis or cardiac amyloidosis. There was also no evidence for myocardial fibrosis or acute/chronic inflammatory remodeling of the pericard.

## Discussion

This successful treatment of a fulminant COVID-19 course with poor prognosis indicates that the use of CRP apheresis should be considered a therapeutic option in moderate to severe COVID-19 courses with a pronounced increase in CRP plasma levels. Specifically, in patients belonging to the high-risk group because of pre-existing conditions. When initiated in the early phase, CRP apheresis could prevent the deterioration of the patient to mechanical ventilation or to the ICU. Patients who generate more than 100 mg/l CRP otherwise invariably end up on ventilation with 30% mortality (7). The reported patient did not have to be admitted to the hospital and was treated as an outpatient despite a rather low blood oxygen saturation and his pre-existing diabetes type 2.

The described therapy option is not tailored specifically to treat COVID-19, but can be used in every condition that is marked by a sharp increase in CRP levels and in which CRP contributes to accelerated tissue destruction (28). CRP apheresis in this patient efficiently reduced CRP levels even in the initial high synthesis phase. After two apheresis sessions, CRP levels peaked at ~130 mg/l 7 days after the positive antigen test despite CRP depletion on the two past days and then gradually declined with the substantial support of two subsequent apheresis procedures. We can only hypothesize how high CRP levels would have increased without apheresis. The initial rise and numerous other reports support the assumption that CRP would have risen to > 300 mg/l in this patient without this CRP apheresis (7, 10, 12). The patient also received 6 mg dexamethasone from day 7 onwards (after the third apheresis), despite being contraindicated in nonventilated patients according to the guideline. Although it is established that this treatment affects CRP levels by inhibiting the inflammatory response overall, this effect usually needs several days and is not fast (29). In fact, the rationale for the use of dexamethasone was to curb a potential, renewed rise in CRP the following week. The pronounced reduction of CRP during treatments and the overall achieved decrease in the area under the concentration curve should be attributed to CRP apheresis.

The severe and often fatal course of COVID-19 is not caused by the virus itself, but by a massive and excessive immune response, responsible for tissue destructive processes (30). The often-described cytokine storm presents a major responsibility for ARDS and CRP has been established as one of the key markers of this phenomenon, no matter of the underlying disease (22, 31–33). Reducing the decisive factor CRP may cause beneficial effects and slow down immunological self-destruction of the lung and other organs.

CRP apheresis has been used once before in a COVID-19 patient, who was already ventilated and progressed far during the deleterious course of SARS-CoV-2 infection (34). Here, CRP apheresis potentially improved laboratory parameters indicating failure of the heart, kidney and liver. However, CRP plasma levels already reached > 200 mg/l before 1^st^ apheresis and treatment was probably started too late and interrupted for four days to save the patient.

CRP obviously mediates important immune defense mechanisms, which should not be suppressed during an ongoing bacterial infection. However, in this patient who was free of a bacterial infect, CRP levels never declined below 10 mg/l during the apheresis treatment. Rather, the dramatic rise in CRP concentration, mediating pathological tissue destroying functions, was inhibited by CRP apheresis.

Experimental therapies should be realistically considered, especially in the current situation where there are still no satisfying specific therapies for COVID-19 patients and where it is just becoming apparent what the long-term consequences (long COVID) will be in recovered patients.

Cyclooxygenase inhibitors, antibiotics, corticosteroids and other anti-inflammatory therapies do mildly affect CRP as a side effect (35, 36). However, these therapies needed several days in order to affect CRP levels. This is not feasible in the setting of an acute phase response, in which CRP levels rise dramatically within a day or two and need to be reduced in a short time window after the initial rise of the CRP amount. CRP apheresis is selective and efficient and only affects the negative effects of high CRP concentrations but not the rest of the inflammatory response.

While this patient successfully improved with CRP apheresis, a definitive cause and effect relationship needs to be analyzed in a larger group of patients. A randomized clinical trial using CRP apheresis in early-phase COVID-19 patients is planned. For now, CRP apheresis can be considered a safe and potentially effective treatment in patients with a moderate to severe COVID-19 course, specifically when started right after the initial increase of plasma CRP levels or early after the start of ventilation.

## Patient Perspective

Through my daily work, I have already known about CRP apheresis and its positive effects. The theoretical and clinical approach for the treatment of my diagnosed COVID-19 was comprehensible to me and I was very happy that my colleagues treated me and averted a bad course. I had a good feeling throughout the therapy.

## Data Availability Statement

The original contributions presented in the study are included in the article/supplementary material. Further inquiries can be directed to the corresponding author.

## Ethics Statement

The patients/participants provided their written informed consent to participate in this study. Written informed consent was obtained from the individual(s) for the publication of any potentially identifiable images or data included in this article.

## Author Contributions

AS drafted the manuscript. JR and CB reviewed and edited the manuscript. JR, AR, and CB collected all data. AR, CB, and AS coordinated CRP apheresis. All authors contributed to the article and approved the submitted version.

## Conflict of Interest

AS is Founder and Shareholder of Pentracor GmbH. CB and AR are employees of Pentracor GmbH.

The remaining author declares that the research was conducted in the absence of any commercial or financial relationships that could be construed as a potential conflict of interest.

## Publisher’s Note

All claims expressed in this article are solely those of the authors and do not necessarily represent those of their affiliated organizations, or those of the publisher, the editors and the reviewers. Any product that may be evaluated in this article, or claim that may be made by its manufacturer, is not guaranteed or endorsed by the publisher.
